# Advances of Area-Wise Distributed Monitoring Using Long Gauge Sensing Techniques

**DOI:** 10.3390/s19051038

**Published:** 2019-02-28

**Authors:** Liming Zhou, Jian Zhang

**Affiliations:** 1 Jiangsu Key Laboratory of Engineering Mechnics, Southeast University, Nanjing 210096, China; 2 School of Civil Engineering, Southeast University, Nanjing 210096, China; liming_zhou@seu.edu.cn

**Keywords:** area-wise distributed monitoring, long gauge sensing, structural health monitoring

## Abstract

This paper provides an overview of the area-wise distributed monitoring based on long gauge sensing to meet the requirements in the field of structural health monitoring (SHM), the methodology is reviewed and its application is discussed in this paper. First, a long gauge sensing technique developed for SHM, which utilizes carbon fiber and optical fiber sensors with important technical improvements is introduced and described. Second, area-wise distributed monitoring is discussed in order to demonstrate the high-performance of this approach in structural monitoring using a network of long gauge sensors. Third, theories of processing area-wise distributed monitoring data for comprehensive structural identification have been developed, which perform a rich recognition of local and global structural parameters including structural deflections, dynamic characteristics, damages, and loads. This area-wise distributed monitoring concept and the aforementioned long gauge sensing technique are finally embedded into an SHM system to offer a viable monitoring solution for groups and networks of infrastructural systems. Some successful applications are cited to confirm the effectiveness of the SHM system.

## 1. Introduction

Over the past two decades, structural health monitoring (SHM) systems have been devised, implemented worldwide, and continue to evolve, improve, and monitor the structural performance and operational conditions of various types of engineering structures under their in-service life [1,2,3,4,5,6]. The overall purpose of a SHM system is to monitor the in-situ behavior of a structure accurately and efficiently to determine its health or physical condition [7,8,9]. A successful structural health monitoring strategy is one that is able to promptly and accurately examine the status of the integrity of a structure using embedded or attached sensors, and intelligently utilize the data to assess the state of the structure [10,11]. A structural health monitoring system is typically comprised of two essential components, (a) a sensor network system consisting of data acquisition, communication, data processing, storage, and management; and (b) a post processing system achieving the function of data analysis and interpretation, structural identification, local and global diagnosis, damage detection, and residual life prediction [12,13,14].

While the field of SHM has witnessed significant advancements over the past two decades and a wide range of SHM methodologies has been introduced and implemented, there still remain challenges to be addressed in the areas of sensing, as signal processing, diagnostics, and analysis methods which hinder the wide application of SHM in engineering practices. For instance, it has been well recognized that the existing SHM systems still have difficulty to identify some structural damages, especially minor damages, such as early corrosion. This is due to the fact that there are two bottlenecks in currently used intelligent sensing techniques. These include:

(1) Structural damages cannot be effectively analyzed since the current sensing technology has shortcomings in dealing with massive data. The biggest challenge is the lack of a sensing technology tailored specifically for civil engineering structures and for effective structural performance evaluation based on monitored data. Measurement information produced by various types of strain gauges (electronic strain gauges, fiber FBG sensors, etc.) is too local. For instance, their ability of capturing cracks in huge civil structures is analogous to searching for a needle in a haystack. Moreover, various types of accelerometers and displacement meters are too macroscopic. Measurement signals along with the information they contain, such as frequency, have weak correlation with structural damage. Overall, when a large number of different types sensors collect a large volume of diverse data—such as strain, temperature, acceleration, etc.—minor structural damages cannot be easily detected. Utilization of natural frequency analysis from acceleration sensing for detecting crack damage, as reported by Farrar [15], demonstrated that artificially cutting a steel bridge did not result in the reduction of the natural frequency, as expected, but on the contrary in an increase in the measured natural frequency in the case of inducing minor damage. This frequency change is only 6.9% in the case of serious structural damage. Liu and DeWolf [16] tested the natural frequency of an actual bridge for a year, the results of their study showed that structural natural frequency suffered great dispersion due to the effect of temperature and other reasons, and the change due to temperature was even more pronounced than the effect of structural damage on natural frequency. Overall, using the changes in the natural frequency, measured based on acceleration measurement—as a means for damage identification—is difficult, especially during the early stage of damage.
(2) The sensor monitoring device has inadequate comprehensive performance: single function or single performance, and poor durability. First, existing sensors have mostly a single function and a wide range of monitoring indicators. Commonly used global structure sensors include accelerometer, gyroscopic sensors, inclination, and a few other types. Through using these sensors structural acceleration, displacement, rotational deformations, and other macro indicators and responses can be measured and monitored. Commonly used local sensors include devices such as strain gauges, or point type optical fiber sensors, that can be used to monitor, detect, and measure damage—such as cracks, corrosion, etc.—in structural details such as joints and connections. Numerous types of sensor networks require various types of sensors, which lead to the complexity and high cost of SHM systems [2]. Second, the existing sensors have a single performance function and lack comprehensive performance. For example, fiber Bragg grating (FBG) strain sensing and Brillouin scattering sensing are two kinds of commonly used optical fiber sensing tools [17,18,19]. Due to its small size and light weight, FBG strain sensor achieves high accuracy by being directly attached to the surface of structures, however, it lacks the ability of distributed damage coverage. Brillouin scattering sensing is a nominally distributed sensing. It is capable of essentially sensing a dense distribution of point strain, which is obtained by backscattering weighted over a certain distance. Therefore, local damage is easily submerged. In addition, the Brillouin scattering sensors have the problem of low precision and low sensing speed. Third, the above two types of sensors have the problems of fragility, slippage, and non-durability, which negatively influence their precision, sensitivity, and long-term performance in engineering applications [20].

In order to overcome the aforementioned problems and challenges, associated with whole sensing and local sensing in civil engineering structures, two types of sensing technologies have been proposed, including large-area gauge sensing [21,22,23,24,25,26,27] and long-gauge sensing [28,29,30,31,32]. Both sensing techniques have achieved damage coverage by expanding the area and length of the sensor respectively, and their effectiveness has been proven in practical engineering. Kong et al. [21,22] proposed a large-area strain sensing technology, which is based on a soft elastomeric capacitor and can long-term monitor fatigue cracks in steel bridges. Yao and Glisic [23,24] designed a sensing sheet based on large area electronics and used the sensors to successfully detect the propagation of fatigue cracks based on the time history of the measured strain. In this paper, we will introduce area-wise distributed monitoring based on long gauge sensing for a structure or structure groups.

The cost of the SHM system based on long gauge sensor is relatively low. A typical SHM system is mainly composed of a hardware system, such as sensors and data collection/acquisition instruments, and a software system embedded with diverse signal processing algorithms. Although the traditional sensors—for instance, point type strain gauge—can partially achieve some purposes about measuring the responses of monitored structures, it is extremely unpractical because due to the large scale of general infrastructures, a dense installation of this kind of sensors is required but the budget is limited. Moreover, for electrical resistance based sensors that are extensively used in smart structures, more sensor means more cable to connect, which causes another application barrier, electromagnetic interference. Unlike the traditional health monitoring systems which builds the structural information by acquiring structural responses such as displacement, acceleration, and point type strain, the reviewed long gauge sensing technology in this paper has the capability of multi-functional direct mapping, which simplifies the health monitoring systems. The long gauge strain sensor has a single sensing mechanism, but it is capable of performing multiple monitoring functions, including deformation, angular, load, and so on. Therefore, the new health monitoring system can effectively reduce costs. Due to the excessive number of nodes and components in large and complex structures, it is impossible to deploy sensors in all locations in engineering practice. Optimizing the number and location of sensors through area-wise distributed strategy can not only effectively reduce the cost of monitoring, but also improve the efficiency of information processing adopting the monitoring system. Thus, it is of great significance. Here, the SM130 Optical Sensing Interrogator was used for strain measurements, which is produced by Micron Optics, Inc. More information concerning the SM130 interrogator the readers can refer to the product introduction on the official website. Overall, the SHM system based on area-wise distributed monitoring and long gauge sensing techniques is inexpensive.

The objective of this paper is to review the state-of-the-art of long gauge sensing techniques, and the related structural identification concepts, as well as its applications in engineering practices. The structure of this paper is as follows: In Section 2, long gauge sensing is introduced to meet the needs of SHM, and the long gauge sensing technique utilizing carbon fiber sensors, optical fiber sensors, and their applications are proposed. In Section 3, the area-wise distributed monitoring is presented for effective monitoring of large-scale structures. Section 4 elaborates a comprehensive structural identification concept based on long gauge strain data and its applications, which consists of macro deformation calculation, long gauge strain modal analysis with derived global modal properties, damage identification, and load identification. In Section 5, the concept of area-wise distributed monitoring in SHM and its application in a case study associated with shinkansen in Japan is presented. Finally, conclusions of this study are drawn.

## 2. Long Gauge Sensing Technology

**Figure 1:**
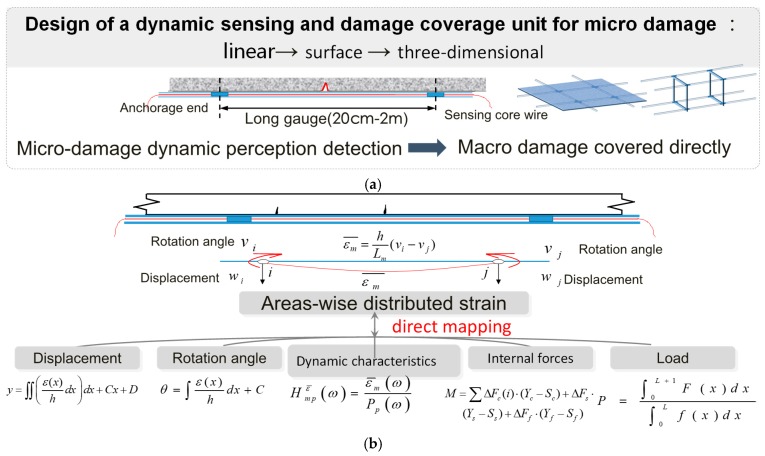
The unique characteristics of area-wise distributed monitoring. (**a**) Macro/micro combination, damage cover unit. (**b**) Direct mapping relationship between area-wise distributed strain and structural parameters.

As discussed above, it is apparent that new sensing technologies need to be developed for the successful implementation in SHM which has many unique characteristics. On the one hand, the concept of SHM requires that the utilized sensing tool has the capability of macro/micro damage coverage [33,34,35,36,37]. Traditional strain gauges deployed in an area of a structure may output many different strain values because they are sensitive to local information such as cracks. Thus, multiple strain gauges are generally used to get the average strain within the crucial area. Hence, the concept of long gauge sensing is proposed to lessen this difficulty [38,39,40,41,42]. By designing a gauge length usually between 20 cm and 200 cm, the long gauge strain sensor can monitor average strain in a relatively large area and it is more likely to detect cracks [43,44,45]. As shown in Figure 1a, regionally distributed sensing can be carried out through assembling different types of long gauge strain sensors in forms of linear, surface (mesh), three-dimensional, or other types of distribution in order to achieve the SHM targeted at key regions of a structure. On the other hand, the long gauge sensing technology has the capability of multi-functional direct mapping. Besides monitoring structural local information, the long gauge sensors can extract global structural information as well. As shown in Figure 1b, the strain output from the long gauge sensor has a direct relation with rotational angle, which is expressed as follows: $\bar{\varepsilon }=\left(\theta_{i}-\theta_{j}\right)h/L$ where $\bar{\varepsilon }$ refers to long gauge strain, $\theta_{i}$ and $\theta_{j}$ refer to the rotational angles at two ends of the long gauge sensor respectively, h refers to the distance from the sensor location to the neutral axis of the beam section, L refers to the gauge length [46,47]. There is a direct relationship among output values of long gauge strain, displacement, angular, load, and other parameters for the area where the area-wise distributed strategy is selected for monitoring. In the meantime, through combining the method that will be introduced in this paper, we can develop a long gauge sensor that can function as an accelerometer and other types of traditional sensors. This means the proposed design introduces a novel sensing system, which is a single sensing mechanism, yet it is capable of performing multiple monitoring functions, including acceleration, strain, angular strain, deformation, load, internal force, and damage detection.

### 2.1. Long Gauge Carbon Fiber Sensor

**Figure 2:**
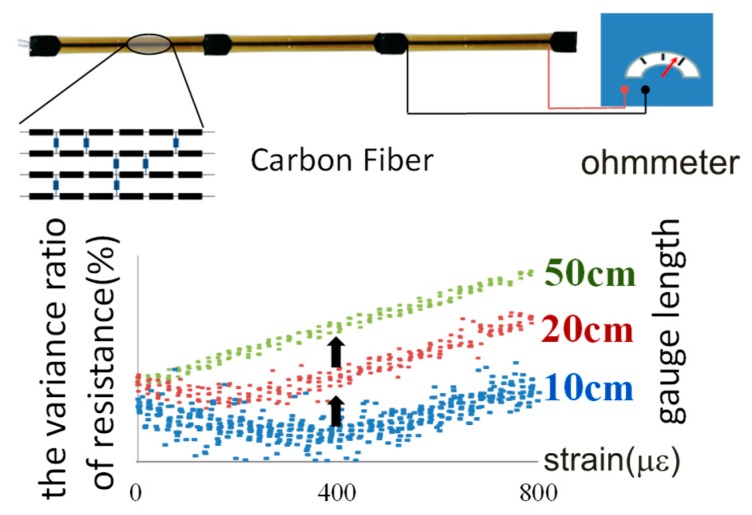
Techniques for long gauge carbon fiber sensing.

To carry out the long gauge sensing technology described above, a long gauge carbon fiber sensor was developed. The axial deformation of carbon fiber composite along its fiber direction leads to the variation in electrical resistance. This makes it possible to accurately determine small strains. Two strategies have been developed to enhance the behavior of the carbon fiber sensor [48,49]. First, by increasing the gauge length, the linear performance of the standard strain against the electrical resistance can be enhanced, as shown in Figure 2. Thus, the capacity to monitor tiny strains is improved. Second, by cutting an incision in the sensing core, along its fiber direction, a magnified stress area will appear and it enhances the sensitivity of the sensor.

### 2.2. Long Gauge Fiber Bragg Grating (FBG) Sensor

**Figure 3:**
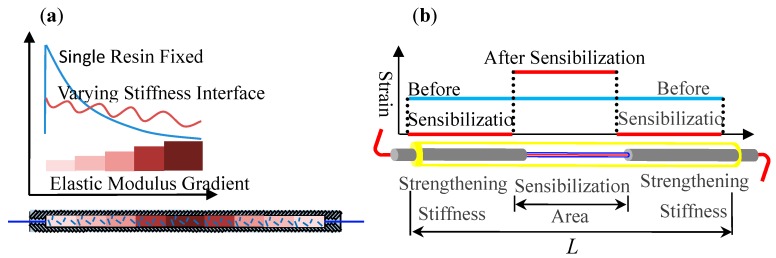
Methodology for the long gauge optical fiber sensing: (**a**) Anchoring; (**b**) Sensibilization.

**Figure 4:**
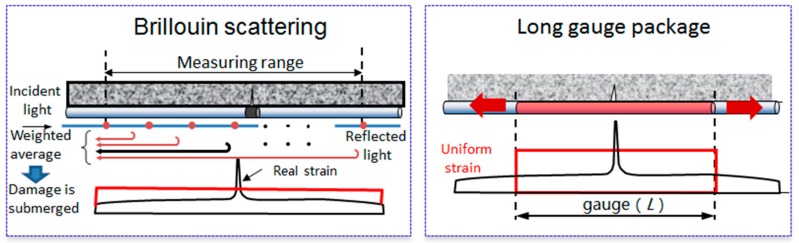
Technique of the long gauge Brillouin scattering sensing: (**a**) Brillouin scattering sensing; (**b**) Long gauge package.

In addition to the long gauge carbon fiber sensor described above, a long gauge FBG sensor is also designed with the encapsulation technique, as shown in Figure 4, to ensure its high accuracy, sensitivity, and long-term performance [50,51]. First, a method for a lightly-loaded anchorage is developed by implementing varying stiffness in the bonding interface between the optical fiber and the resin as shown in Figure 3a, in which the anchoring stress is distributed over the anchorage length and prevents stress concentration. Meanwhile, short fibers in the anchorage zone improve the anti-fatigue and anti-creep capabilities thus, ensure a durable and reliable anchoring of the optical fibers. Second, sensitivity enhancement is achieved by changing the stiffness along the gauge length as shown in Figure 3b. The modification of varying stiffness along the gauge length enables the sensor to amplify the strain in the grating area when both ends of the sensor are fixed securely, which further results in more than 400% increase of strain sensibility. In addition, a highly durable and self-adaptive fiber enhancement is achieved for protective encapsulation by using a fiber composite material which has an approximated thermal expansion coefficient to the optical fiber.

### 2.3. Long Gauge Brillouin Scattering Sensor

Although the Brillouin scattering is a nominally distributed sensing, it is also composed of infinite number of dense point distributions. The strain at each point is obtained by weighting the backscattering of a certain spatial distance as shown in Figure 4a. Therefore, the non-uniformity strain seriously affects the transmission perceptibility and local damage can be easily submerged. Thus, a long gauge Brillouin scattering sensor with the encapsulation technique is also developed [52,53,54,55,56], which not only overcomes the problem of the submerged local damage but also increases the long-term accuracy by more than 5-fold (from ±25 με to ±5 με), shown in Figure 4b.

**Figure 5:**
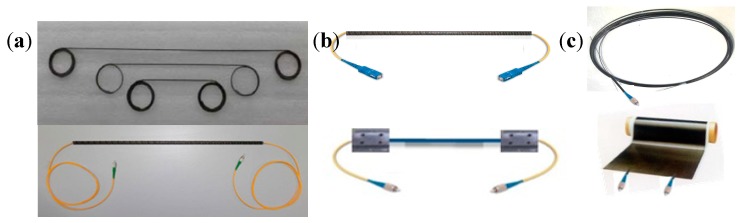
Gauge sensors: (**a**) long gauge optical fiber sensors; (**b**) embedded anchorage and fixed anchorage (from top to bottom); (**c**) self-sensing cable and self-sensing cloth.

The long gauge sensing technology can reveal both micro and macro structural information when compared with traditional point strain sensing. Explicit technologies like fiber encapsulation, varying stiffness anchoring, and sensibilization help long gauge sensing’s applications in optical fiber, carbon fiber, and self-sensing FRP members achieve high precision and good durability. Figure 5 displays the products of long gauge sensing technology including optical fiber sensors, different anchorage methods, and self-sensing FRP members, which are produced by the author’s team. The long gauge sensing technology further lays foundation for the concept of areas distributed sensing and the SHM system for infrastructural groups, which will all be described in the next sections.

## 3. Area-Wise Distributed Monitoring

The objectives and the purposes of structural monitoring have to be established according to the requirements set forth by the asset management authorities, emanating from the expectations from public, the owner and the users. The basic requirements for structural health monitoring can be summarized in the followings: (i) It can accurately and efficiently monitor various health indicators, including strain, deflection, rotation angle, modal parameters, damage, load, etc. (ii) It can identify various types of damage and accurately grasp the ‘mental state’ of the structure. (iii) It can predict the structural life expectancy. In order to address the aforementioned requirements and challenges in monitoring of civil engineering structures, the concept of area-wise distributed sensing and monitoring for a structure or structure groups is presented in this paper.

**Figure 6:**
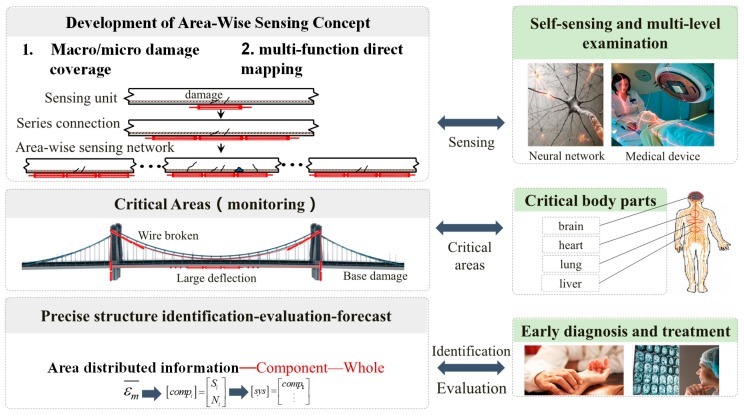
Concept of area-wise distributed sensing and monitoring of a structural system.

In many respects, structural health monitoring is analogous to monitoring the health of human body (Figure 6). Both human body and engineering structures have key components. The nervous system in human body is comprised of a central system, or the command center, consisting of the brain and the spinal cord. In addition, there is a peripheral nervous system which includes a network of nerves that connects the rest of the body to the central system. This complex sensing system collects information from inside the body and from the environment outside the body—such as heat, taste, noise, etc.—and processes the collected information and then dispatches instructions to the rest of the body, facilitating an appropriate response. For smart structures the arrangements and the number of sensing units are limited and, comparatively speaking, will never be able to reach the magnitude or the effective distribution, or facilitate the response, that would mimic the human body and its nervous system. Although the human body’s nervous system is complex, the human body has a few major organs—such as heart, liver, lung, kidney and others—whose health status indicates if the state of the body is safe, functions properly, and conducts all normal human activities and expected behaviors. It is always a priority to monitor and maintain the health of these vital organs whose health and safety assures the overall health of human body. Similar to human body, civil engineering structures are complex, however, they also have key components and critical areas whose health and safety assures the safety of the structures. Therefore, monitoring these critical areas is similar to monitoring the major organs in a human body, in order to assure the safety and health of a structure. These areas which can easily suffer structural damage, and play an important role in structural performance, are known as critical areas. If distributed sensing systems are placed in these critical areas (area-wise distributed sensing), through dynamic and static, macro and micro monitoring, and multi-level analysis structural damage and potential failure can be detected with more accuracy, and hence the distributed sensing systems will result in early warning. Subsequently, a comprehensive identification of structural state parameters, damage state and loads, and direct evaluation and prediction of structural performance can be achieved.

Based on the unique advantages of long gauge sensors, the basic principle of area-wise distributed monitoring from long gauge units to region composition to overall mesh formation is established. Then, the method of determining the critical region size and unit length of the structure is determined, and the method for the recognition of the entire structure based on the key region is implemented. The main procedure for the implementation of this concept is as follows. First, to identify sections prone to damages using structural vulnerability analysis. Originating from earthquake engineering, vulnerability analysis [57] refers to conditional probability of structural damage under certain seismic intensity. Once the potential location of damage is determined, the size of the key structural areas—namely the size of the areas to be potentially damaged, and possibly influenced areas nearby—can be determined based on reliability analysis [58,59,60]. Structural reliability is defined as to determine the ability of the structure or its component against failure given the time and the loading condition. Since, in reality, there is randomness presented in structural materials, dynamic characteristics, and response, it is reasonable to assess the structure’s performance with probability-based reliability analysis. Second, to connect key areas and some general areas into a distributed network by long gauge sensors. Subsequently, to measure macro and micro structural information in an accurate and durable manner. Third, conduct multi-level data analysis not only to gain insight into the behavior of the key structural areas, but also to deduce the information for non-monitored areas via linear interpolation. Therefore, the entire structure is effectively covered by area-wise distributed monitoring.

## 4. Theory and Application

The methodology of area-wise distributed monitoring using long gauge sensors is a systematic SHM framework which consists of sensing and monitoring and identification. Long gauge sensing and area-wise distributed monitoring techniques are able to accurately and durably obtain both micro and macro structural information. The following identification concept developed by the authors and presented herein can reveal a wide range of structural properties such as deformation, dynamic characteristics, damages, and loads.

### 4.1. Macro Strain Modal Theory

Due to the unconventional characteristics of long gauge sensing and area-wise distributed monitoring, the long gauge modal strain theory [35,61,62] has an overwhelming superiority to traditional modal acceleration theory. Specifically, the proposed modal strain theory not only fulfills the functions of modal acceleration theory to extract global modal properties, but also surpasses that approach with respect to its ability in detecting local damage.

The displacement FRF obtained from structural acceleration response and the long gauge strain FRF are listed as

(1) $$H_{lp}^{d}\left(\omega \right)=\sum_{r=1}^{N}\frac{\varphi_{lr}\varphi_{pr}}{M_{r}(\omega_{r}^{2}-\omega^{2}+2j\xi_{r}\omega_{r}\omega )}$$

(2) $$H_{mp}^{\bar{\varepsilon }}\left(\omega \right)=\sum_{r=1}^{N}\frac{\eta_{m}(\varphi_{ir-}\varphi_{jr})\varphi_{pr}}{M_{r}(\omega_{r}^{2}-\omega^{2}+2j\xi_{r}\omega_{r}\omega )}$$

where $H_{lp}^{d}$ is the displacement FRF between the excitation at the pth node and the displacement response at the lth node, $\varphi_{lr}$ refers to the mode shape coefficient of the rth mode at the lth node, $M_{r}$ is the rth modal mass, $\omega_{r}$ is the rth modal frequency, $\xi_{r}$ is the rth modal damping ratio, $H_{mp}^{\bar{\varepsilon }}$ is the long gauge strain FRF between the excitation at the pth point and the long gauge strain response at the mth unit, *i* and *j* are the two end nodes of the mth unit. $\eta_{m}=\frac{h_{m}}{L_{m}}$ where $L_{m}$ is the gauge length of the sensor and $h_{m}$ is the distance between the sensor and the neutral axis of the section.

By comparing the two FRFs, several conclusions can be made: First, the long gauge strain FRF can calculate the same modal frequency $\omega_{r}$ and modal damping ratio $\xi_{r}$ as the displacement FRF, because the two FRFs share the same denominator, with the same modal frequency and modal damping ratio values. Second, the long gauge strain FRF can be used to derive the displacement mode shape. Though the eigenvectors of the two FRFs are different, their mathematical relationship is the same as the mathematical relationship between the long gauge strain and displacement. Thus, it is convenient to derive displacement mode shapes from the long gauge strain mode shapes. Third, the long gauge strain FRF has the same phase as the displacement FRF. Fourth, the long gauge strain FRF is more similar to the displacement FRF than velocity FRF or acceleration FRF, and it is more sensitive to the response in low frequency. So it is suitable for using with highly flexible structures such as skyscrapers, long span bridges, and cables.

**Figure 7:**
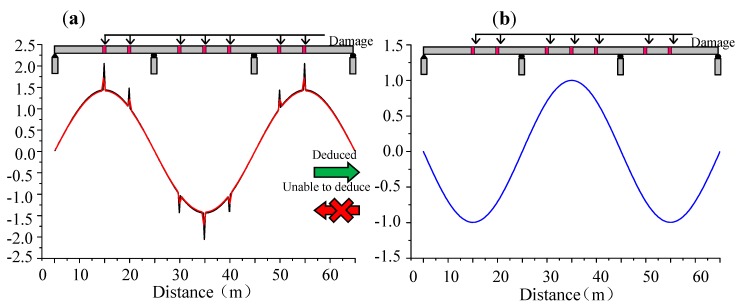
Advantage of the long gauge strain modal theory [63]. (**a**) Strain Mode Shape. (**b**) Displacement Mode Shape.

As mentioned above, the long gauge strain modal theory can obtain accurate global modal properties such as frequencies, mode shapes, damping ratios, and phases, which fulfill the same functions of modal acceleration theory. However, modal acceleration theory has some limitations in identifying long gauge strain modal information. For instance, long gauge strain mode shapes cannot be precisely calculated by displacement mode shapes while displacement mode shapes can be successfully obtained from long gauge strain mode shape under 10% noise as shown in Figure 7 [63]. Furthermore, the modal displacement method is too global to effectively detect the local damage. However, the long gauge strain modal concept is very sensitive to local damages based on its measured strain. Several damage identification methods based on long gauge sensing will be described in the following discussions. In general, the long gauge strain modal approach is able to replace the modal acceleration method.

### 4.2. Structural Damage Identification

Although the concept of damage identification based on acceleration measurement has been developed for a long time [64,65,66], it is insensitive to local damages. On the other hand, the damage identification based on point strain is able to detect local damages, the gauge length of the point strain sensors is too short to effectively cover cracks. On the contrary, long gauge strain data from area-wise distributed monitoring includes the local structural information directly and the long gauge can cover a relatively large area. Thus, consequently, it has a better identification ability for damage identification. The following discussion elaborates three effective damage identification methods based on area-wise distributed monitoring.

#### 4.2.1. Crack Width Monitoring

**Figure 8:**
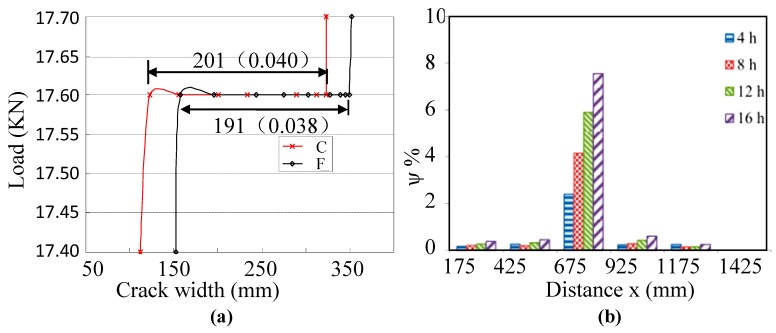
(**a**) A comparison of average crack width between long gauge FBG [37]; (**b**) Corrosion levels along the length of the beam based on CSR [67].

Reinforced concrete bridges usually have cracks caused by various complicated factors such as overload, shrinkage and creep. The occurrence of cracks decreases the structural stiffness leading to the excessive deflection of structures. Therefore, crack monitoring is important for the bridge’s maintenance. However, it is difficult to monitor cracks online and continuously by utilizing traditional resistivity-based crack transducers or extensimeter. Herein, a method for long term crack width monitoring is developed based on long gauge sensing technique. The average crack width is approximately equal to the product of the long gauge strain and the gauge length: ω≈ε×L, where ε is the long gauge strain and L is the gauge length. A validation between 200 mm long gauge FBG sensors (F) and traditional crack transducers (C) is shown in Figure 8a. The results show good agreement and prove the effectiveness of the proposed crack width monitoring method [44].

#### 4.2.2. Corrosion Inspection

Corrosion of steel reinforcements has been the focus of a large number of research studies in recent years, due to its adverse impact leading to early deterioration and shortening of the service life of concrete structures, especially those prone to salty and acidic environment. A new technique for the corrosion monitoring of reinforcing bars in flexural reinforced concrete structures is introduced. This method uses distributed embedded long-gauge packaged carbon fiber line (PCFL) sensors to realize self-compensation under working load. Two different methods of continuous-strain ratio (CSR) and distributed-strain ratio (DSR) are used to evaluate the corrosion levels [67].

The purpose is to monitor the progress of steel corrosion under a working load. The corrosion level values of each section were calculated using DSR method. Figure 8b illustrates the calculated corrosion levels along the beam by using the measured distributed strains of PCFL. It can be clearly observed that the locations of corrosion damage can be clearly and visibly found by using this method.

#### 4.2.3. Damage Identification

During the past few decades, several model-free vibration-based damage identification methods have been developed utilizing various dynamic characteristics such as modal stiffness, modal strain energy, natural frequencies, mode shapes and their derivatives, modal flexibility and its curvature, frequency response functions and their curvature, and power spectral density. These techniques have received wide attention because they are simple, fast, and inexpensive [36]. As discussed above, measurement information produced by various types of traditional sensors are too local or too macroscopic, and are insensitive to the damage. The distributed strain measurement techniques are more sensitive to perturbations in vibration characteristics. Many algorithms might still remain as viable tools for practical damage identification. However, their performance can be enhanced by distributed strain sensing measurement techniques. It has been well accepted that the strain mode shape is an excellent index for structural change localization [68]. Thus, it is a suitable index to be used for the deduction between the mode shapes at intact condition and damaged condition.

**Figure 9:**
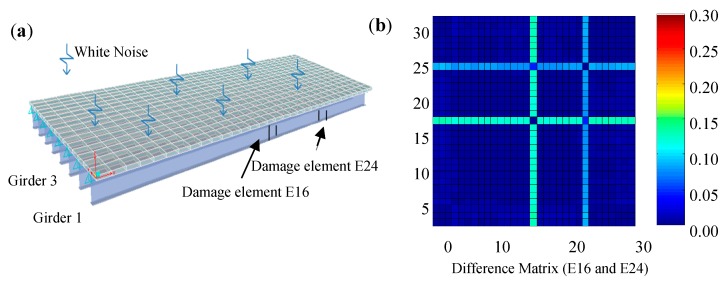
Damage identification of a steel stringer bridge: (**a**) Damage details; (**b**) Damage index of difference matrix [68].

A finite element model of a simply supported steel girder bridge is considered as an example as shown in Figure 9. Other than the intact structure, a damaged structure is also simulated, in which 10% stiffness loss are made on the elements 16 and 24 of girder 1 as shown in Figure 9a. By estimating strain FRFs from simulated ambient vibration data with 10% white noise and picking the peaks of the strain FRFs in a column in the first mode, the difference matrices of strain mode shapes of the intact and the damaged structures are calculated as shown in Figure 9b. As can be seen, this approach can clearly locate the structural damage.

### 4.3. Structural Identification

#### 4.3.1. Structural Deflection Identification

Due to limitations in practical applications, existing contact or non-contact deflection transducers rarely meet the need for the deflection measurement in the SHM of long span bridges. Combining the distributed long gauge sensing technique and classical conjugate beam method, this paper recommends a solution to measure the displacement and the rotational angle [69]. The basic idea is to transfer the deformation in the real beam into the internal forces in the virtual beam whose shear force and bending moment are equal to the rotational angle and the displacement of the real beam respectively.

**Figure 10:**
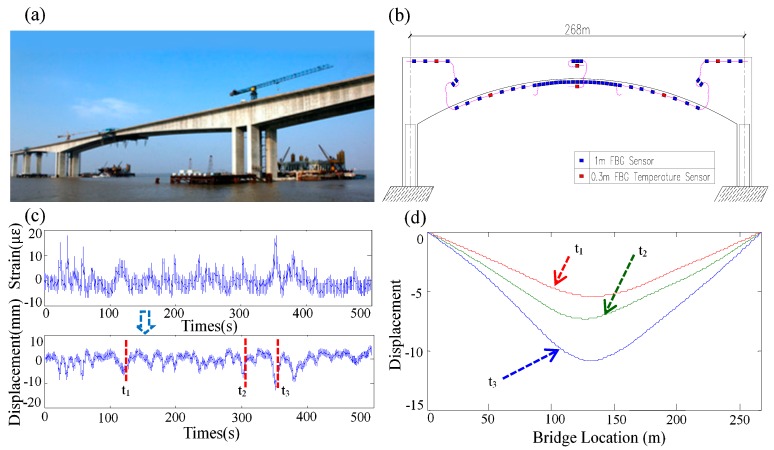
Area-wise distributed monitoring of the Sutong Auxiliary Bridge: (**a**) Bridge Photo; (**b**) Sensor layout; (**c**) measured long gauge strain and identified deflection; (**d**) Deflection distribution [70].

An example for calculating the structural deflection distribution from the measured long gauge strains of the Sutong Auxiliary Bridge is illustrated in Figure 10c. Herein, another example of a post-tensioned concrete box girder bridge is also provided [70]. The bridge has a total length of 191 m, with a main span of 85 m and two side spans of 53 m. (Figure 10a). The sensor layout of the bridge is shown in Figure 10b. The LG-FBG strain sensors were used to measure structural strains, in the ambient vibration test. The works to indirectly calculate structural deflections from measured long-gauge strains are performed and the results are shown in Figure 10d.

#### 4.3.2. Neural Axis Depth Identification

The neutral axis depth (NAD) is an important parameter for structural design and structural safety evaluation. A few approaches have been introduced in the literature for NAD estimation using static or dynamic strain measurements. In this section, a new method to determine the neutral axis position is presented. This approach is based on the concept of modal strain and consists of four main steps:

(i) Acquiring dynamic strain data with the installed strain sensing and data acquisition system;
(ii) Obtaining the peak value at the selected natural frequency of the frequency spectrum, which is calculated from the strain time history via fast Fourier transform (FFT);
(iii) Extracting the coefficient for the position of neutral axis *ψ*, through a linear fitting between the modal strains of two sensors within the same cross section, namely the slope of the fitting line $\psi =\varepsilon \left(\omega_{r}\right)/\varepsilon^{\prime }\left(\omega_{r}\right)$ in which $\varepsilon \left(\omega_{r}\right)$ and $\varepsilon^{\prime }\left(\omega_{r}\right)$ are the modal strain of the rth order mode.
(iv) Extrapolating the position of the neutral axis via equation $h=\frac{\psi }{\psi +1}H$ or $h=\frac{\psi }{\psi -1}H$ using the spatial information of sensor installation.

**Figure 11:**
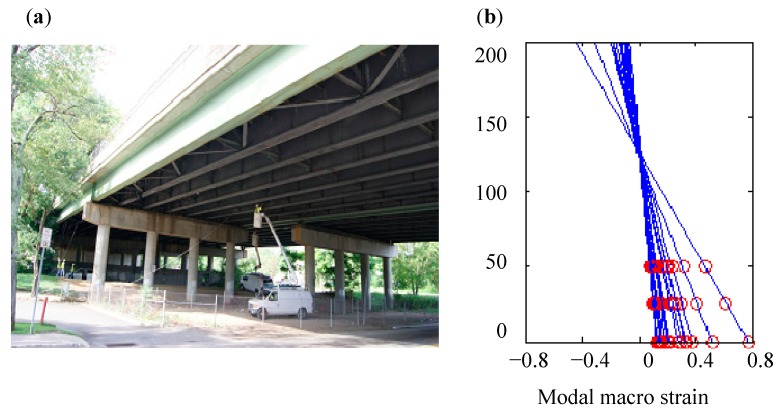
Area-wise distributed monitoring of the multi-girder steel stringer bridge: (**a**) The Investigated Bridge. (**b**) Modal macro strain profile [71].

Neutral axis positions for the girders of a bridge are studied by recording macro strain time histories [71]. The investigated bridge, located in northern New Jersey in US, is a multi-girder steel stringer bridge constructed in 1984. It is comprised of four spans using a standard steel stringer design of girders (Figure 11a). Figure 11b shows the long gauge strain modal profile of the section with sensors 19, 1, and 10, in which three points in each line denotes three long gauge strain modal values recorded by those three sensors. It is seen that the neutral axis positions determined from all datasets are very consistent, even the data sets recorded at different times.

### 4.4. Load Identification

**Figure 12:**
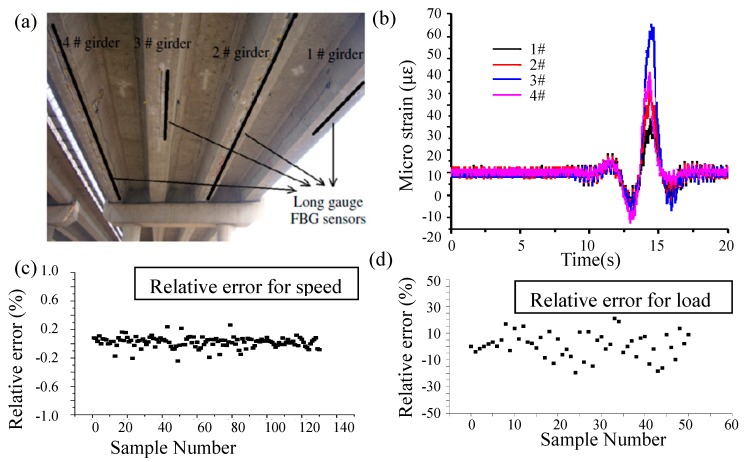
Load application to a continuous beam bridge: (**a**) Sensor installation; (**b**) Typical long gauge strains; (**c**) Relative error for speed; (**d**) Relative error for load [72].

The loading identification method using long gauge strains are studied to measure both load and vehicle speed in a practical and robust manner [72]. Under multi-axle load, the identified vehicle load is expressed as

(3) $$P=\frac{\int_{0}^{l_{1}+L_{2}}F\left(x\right)d_{x}}{\int_{0}^{L_{1}}f\left(x\right)d_{x}}$$

where P is the vehicle load, $L_{1}$ is the bridge length, and $l_{1}$ is the vehicle length, F(x) is the influence line of bending moment at mid-span section under multiple axles, x is the distance between the first axle to the bridge end, f(x) is the influence line of bending moment at the mid-span section under a single axle. The desired vehicle speed is identified by $v=\left(L_{1}+l_{1}\right)/\Delta t$, where Δt is the time period between the time when a vehicle enters and leaves the bridge, which is identified by observing the slope variation of F(x). A prestressed concrete box girder bridge with two traffic lanes is taken as an example to illustrate the effectiveness of the load identification method. The monitoring system using the long gauge strain sensors is shown in Figure 12a. The corresponding long gauge strain time history is shown in Figure 12b. Identified vehicle speed and load were compared with the results from weigh-in-motion system as shown in Figure 12c,d. It is seen that the relative error for the moving weights is within 15% and most errors are within 10%.

## 5. The SHM System and Its Application

The sensing technique and strategy thoroughly described above combining with an area-wise distributed monitoring method for structural identification and performance evaluation can be integrated with a SHM system to reveal hidden flaws in a structural state. Moreover, it allows that a SHM system is designed for infrastructural groups instead of a single structure, and it provides a viable method to weave sensing entity into a smart cyber-physical network, to manage infrastructure at a collective level and to help study regional factors influencing structural performance.

The health monitoring of continuous structural groups and application of the concept of area-wise distributed sensing is introduced herein by considering again the health monitoring for safety and early warning system of a high speed railway infrastructure as an example. The Tohoku shinkansen railway from Tokyo to Aomori in Japan, which is a continuous structural group, with a total length of 736 kilometers, covers a large number of key transportation infrastructures. In order to ensure its safe usage within the anticipated service period, it was necessary to carry out safety monitoring on this system, which—due to its large range—is hard to conduct effectively with commonly used methods. First, based on the concept of area-wise distributed sensing of structural groups, key subgroups covering a wide monitoring area were categorized and divided based on the distance from the sea, geological conditions and so on, with the center of each subgroup as the monitoring hub. Second, monitoring bodies needing for essential concerns were further divided within each subgroup, depending on the factor that which subgroup plays a decisive role on the safe operation of the entire shinkansen. Finally, critical areas were selected from the monitoring body and regionally distributed sensing and monitoring were carried out. At the same time synchronous monitoring in the wide area through a collaborative and coordinated effort with the control center was established. By following this approach, the entire structural group could be effectively monitored. For the continuous structural groups, several sub groups were divided generally along the direction of their length. Strategies for data distribution by acquisition hardware was divided into centralized collection or mixed collection. Data transmission architecture between collection stations and monitoring centers was referred to the tree structure.

The monitoring of shinkansen railway bridge was also considered as an engineering example to illustrate the damage identification method via damage fingerprint [44]. Compared with highway bridges, this reinforced concrete continuous railway bridge has small flexibility and small strain. In order to assess the structural condition, the area-wise distributed monitoring was installed in four key areas on the bridge and the piers were covered by long gauge FBG sensors. After the installation, the structural response to an earthquake was recorded.

**Figure 13:**
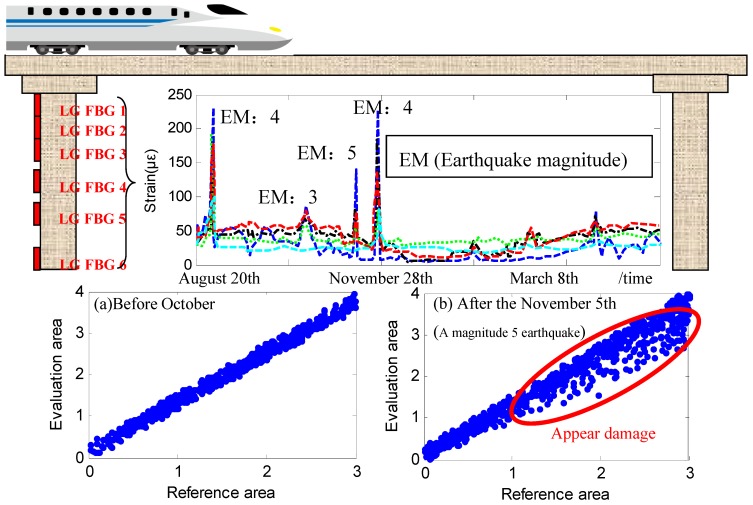
Damage identification of the shinkansen railway bridge by damage fingerprint: (**a**) Before earthquake; (**b**) During earthquake.

The identified structural status before the earthquake is shown in Figure 13a. The identified structural status during the earthquake is shown in Figure 13b. It is clear that the slope of the fitting line, namely the amplitude ratio of strain unit 6 to strain unit 1, has changed, which means the observed earthquake caused damage to the structure. Through field investigation, tiny cracks on the monitoring areas were discovered. The cracks were usually closed unless a train passed by. Obviously the macro indexes such as acceleration and displacement were not sensitive to this damage. The successful discovery of this tiny damage proves the effectiveness of the damage identification method based on long gauge sensing even though the damage is tiny.

## 6. Conclusions

A state-of-the-art methodology called structural area-wise distributed monitoring using long gauge sensing techniques is reviewed in this paper. The main conclusions are as follows:

First, the method of area-wise distributed monitoring provides an effective way for high-precision monitoring of large-scale structures with a limited number of long gauge sensors by connecting key structural areas into a network. In this cost-effective solution, the long gauge sensor not only can effectively reveal both macro and micro information of civil structures, but also has the multi-functional capability of direct mapping and building the relationship among output values of strain, displacement, angular, load, and other parameters.

Second, high precision and good durability of the long gauge sensor were also demonstrated by technical improvements such as adaptive fiber encapsulation, varying stiffness anchoring, which further enables the applications of optical fiber sensors, and carbon fiber sensors in engineering practices.

Third, a comprehensive structural identification methodology, developed by the authors’ team, was also presented. This method is based on processing the long gauge strain measurement. It induces a rich recognition of structural parameters including deflection, crack width, dynamic characteristics, damages, and loads.

Finally, The unique advantages of area-wise monitoring based on long gauge sensing further allows the effective monitoring of infrastructure groups, which enhances the traditional SHM to a new level and offers more research opportunities to explore.

Future research in SHM may focus on the signal processing methods and algorithms capable of handling feature extractions and damage detection in dealing with big data captured from a large number of sensor networks from infrastructural group monitoring systems and the applications of the proposed methodology to a greater variety of engineering cases.

## References

[1] Doebling S.W., Farrar C.R., Prime M.B. (1998). A review of damage identification methods that examine changes in dynamics properties. Shock Vib. Dig..

[2] Chen Z.S., Zhou X., Wang X., Dong L.L., Qian Y.H. (2017). Deployment of a Smart Structural Health Monitoring System for Long-Span Arch Bridges: A Review and a Case Study. Sensors.

[3] Pakzad S.N., Fenves G.L. (2009). Statistical analysis of vibration modes of a suspension bridge using spatially dense wireless sensor network. J. Struct. Eng..

[4] Vitola J., Pozo F., Tibaduiza D.A., Anaya M. (2017). Distributed Piezoelectric Sensor System for Damage Identification in Structures Subjected to Temperature Changes. Sensors.

[5] Siringoringo D.M., Fujino Y. (2008). System identification of suspension bridge from ambient vibration response. Eng. Struct..

[6] Brownjohn J.M.W., Stafano A.D., Xu Y.L., Wenzel H., Aktan A.E. (2011). Vibration-based monitoring of civil infrastructure: Challenges and successes. J. Civ. Struct. Health Monit..

[7] Murayama H., Kageyama K., Ohara K., Uzawa K., Kanai M., Igawa H. Novel measurement system with optic fiber sensor for strain distribution in welded tubular joints. Proceedings of the ASME 27th International Conference on Offshore Mechanics and Arctic Engineering.

[8] Ansari F. (2007). Practical Implementation of Optical Fiber Sensors in Civil Structural Health Monitoring. J. Intell. Mater. Syst. Struct..

[9] Malekzadeh M., Gul M., Catbas N.F. (2012). Use of FBG sensors to detect damage from large amount of dynamic measurements. Society for Experimental Mechanics Series.

[10] Aktan E., Chase S., Inman D., Pines D. Monitoring and managing the health of infrastructure systems. Proceedings of the 2001 SPIE Conference on Health Monitoring of Highway Transportation Infrastructure.

[11] Sohn H., Farrar C.R., Hemez F.M., Shunk D.D., Stinemates D.W., Nadler B.R. (2004). A Review of Structural Health Monitoring Literature: 1996–2001.

[12] Lynch J.P. (2007). An Overview of Wireless Structural Health Monitoring for Civil Structures. Philos. Trans. Math. Phys. Eng. Sci..

[13] Catbas N., Kijewski-Correa T., Aktan E. (2013). Structural Identification of Constructed Facilities: Approaches, Methods and Technologies for Effective Practice of St-Id.

[14] Ou J.P., Li H. (2010). Structural health monitoring in mainland China: Review and future trends. Struct. Health Monit..

[15] Farrar C.R., Baker W.E., Bell T.M., Cone K.M., Darling T., Duffey T.A., Eklund A., Migliori A. (1994). Dynamic Characterization and Damage Detection in the I-40 Bridge over the Rio Grande.

[16] Liu C.Y., Dewolf J.T. (2007). Effect of temperature on modal variability of a curved concrete bridge under ambient loads. J. Struct. Eng..

[17] Meltz G., Morey W.W., Glenn W.H. (1989). Formation of Bragg gratings in optical fibers by a transverse holographic method. Opt. Lett..

[18] Bao X., Webb D.J., Jackson D.A. (1993). 32-km distributed temperature sensor based on Brillouin loss in an optical fiber. Opt. Lett..

[19] Hotate K., Abe K., Song K.Y. (2006). Suppression of Signal Fluctuation in Brillouin Optical Correlation Domain Analysis System Using Polarization Diversity Scheme. IEEE Photonics Technol. Lett..

[20] Lee B. (2003). Review of the present status of optical fiber sensors. Opt. Fiber Technol..

[21] Kong X., Li J., Collins W., Bennett C., Laflamme S., Jo H. (2017). A large-area strain sensing technology for monitoring fatigue cracks in steel bridges. Smart Mater. Struct..

[22] Laflamme S., Cao L., Chatzi E., Ubertini F. (2016). Damage Detection and Localization from Dense Network of Strain Sensors. Shock Vib..

[23] Yao Y., Glisic B. (2015). Detection of Steel Fatigue Cracks with Strain Sensing Sheets Based on Large Area Electronics. Sensors.

[24] Glisic B., Yao Y., Tung S.T.E., Wagner S., Sturm J.C., Verma N. (2016). Strain Sensing Sheets for Structural Health Monitoring Based on Large-Area Electronics and Integrated Circuits. Proc. IEEE.

[25] Hu Y., Rieutort-Louis W.S.A., Sanz-Robinson J., Huang L., Glisic B., Sturm J.C., Wagner S., Verma N. (2014). Large-Scale Sensing System Combining Large-Area Electronics and CMOS ICs for Structural-Health Monitoring. IEEE J. Solid-State Circuits.

[26] Verma N., Hu Y., Huang L., Rieutort-Louis W.S.A., Sanz-Robinson J., Moy T., Glisic B., Wagner S., Sturm J.C. (2015). Enabling Scalable Hybrid Systems: Architectures for Exploiting Large-Area Electronics in Applications. Proc. IEEE.

[27] Hu Y., Huang L., Rieutort-Louis W.S.A., Sanz-Robinson J., Sturm J.C., Wagner S., Verma N. (2014). A Self-Powered System for Large-Scale Strain Sensing by Combining CMOS ICs With Large-Area Electronics. IEEE J. Solid-State Circuits.

[28] Geiger H., Xu M.G., Dakin J.P., Eaton N.C. (1995). Multiplexed measurements of strain using short and long gauge length sensors. Proc. SPIE Int. Soc. Opt. Eng..

[29] Spillman W.B., Huston D.R., Wu J. (2001). Seismic event monitoring using very long gauge length integrating fiber optic sensors. Distributed Fiber Optical Sensors & Measuring Networks.

[30] Liang Y., Tennant A., Jia H., Xiong X., Ansari F. (2005). Implementation of Long Gauge Fiber Optic Sensor Arrays in Civil Structures. Sensing Issues in Civil Structural Health Monitoring.

[31] Li S.Z., Wu Z.S. (2010). Parametric Estimation for RC Flexural Members Based on Distributed Long-Gauge Fiber Optic Sensors. J. Struct. Eng..

[32] Jr W.B.S., Huston D.R. (1996). Pattern detection through the use of long-gauge length spatially weighted fiber optic sensors. Proc. SPIE Int. Soc. Opt. Eng..

[33] Tang Y.S., Ren Z.D. (2017). Dynamic Method of Neutral Axis Position Determination and Damage Identification with Distributed Long-Gauge FBG Sensors. Sensors.

[34] Wu Z.S., Adewuyi A.P., Xue S.T. (2011). Identification of damage in reinforced concrete columns under progressive seismic excitation stages. J. Earthq. Tsunami.

[35] Adewuyi A.P., Wu Z.S. (2011). Modal macro-strain flexibility methods for damage localization in flexural structures using long-gage FBG sensors. Struct. Control Health Monit..

[36] Adewuyi A.P., Wu Z.S. (2015). Vibration-based damage localization in flexural structures using normalized modal macrostrain techniques from limited measurements. Comput. Aided Civ. Infrastruct. Eng..

[37] Li S.Z., Wu Z.S. (2010). A Model-free Method for Damage Locating and Quantifying in a Beam-like Structure Based on Dynamic Distributed Strain Measurements. Comput. Aided Civ. Infrastruct. Eng..

[38] Yuan L., Zhou L., Jin W. (2004). Long-gauge length embedded fiber optic ultrasonic sensor for large-scale concrete structures. Opt. Laser Technol..

[39] Tang Y.S., Wu Z.S. (2016). Distributed Long-Gauge Optical Fiber Sensors Based Self-Sensing FRP Bar for Concrete Structure. Sensors.

[40] Spillman W.B., Huston D.R. (2000). Very long gauge length fiber optic sensing and applications. Proc. SPIE.

[41] Glisic B., Chen J., Hubbell D. (2011). Streicker Bridge: A comparison between Bragg-grating long-gauge strain and temperature sensors and Brillouin scattering-based distributed strain and temperature sensors. Sensors and Smart Structures Technologies for Civil, Mechanical, and Aerospace Systems.

[42] Kim T.M., Kim D.H., Kim M.K., Lim Y.M. (2016). Fiber Bragg grating-based long-gauge fiber optic sensor for monitoring of a 60 m full-scale prestressed concrete girder during lifting and loading. Sens. Actuators A Phys..

[43] Xu B., Liu C.W., Masri S.F. (2009). Modal macro-strain vector based damage detection methodology with long-gauge FBG sensors. Proc. SPIE Int. Soc. Opt. Eng..

[44] Li S.Z. (2007). Structural Health Monitoring Strategy Based on Distributed Fiber Optic Sensing. Ph.D. Thesis.

[45] Zhang H., Wu Z.S. (2008). Performance evaluation of BOTDR-based distributed fiber optic sensors for crack monitoring. Struct Health Monit..

[46] Li S.Z., Wu Z.S. (2007). Modal Analysis on Macro-strain Measurements from Distributed Long-gage Fiber Optic Sensors. J. Intell. Mater. Syst. Struct..

[47] Li S.Z., Wu Z.S. (2005). Characterization of long-gauge fiber optic sensors for structural identification. Proc. SPIE Int. Soc. Opt. Eng..

[48] Wu Z.S., Huang H. (2015). Long Gauge Length Carbon Fiber Strain Sensing Device and Testing Method Therefor. WO Patent.

[49] Fouad N., Saifeldeen M.A., Huang H., Wu Z.S. (2016). Early corrosion monitoring of reinforcing steel bars by using long-gauge carbon fiber sensors. J. Civ. Struct. Health Monit..

[50] Li S.Z., Wu Z.S. (2007). Development of distributed long-gage fiber optic sensing system for structural health monitoring. Struct. Health Monit..

[51] Li S.Z., Wu Z.S. (2009). Sensitivity Enhancement of Long-gage FBG Sensors for Macro-strain Measurements. Smart Mater. Struct..

[52] Yang C.Q., Wu Z.S., Zhang Y. (2008). Structural health monitoring of an existing PC box girder bridge with distributed HCFRP sensors in a destructive test. Smart Mater. Struct..

[53] Tang Y.S., Wu Z.S., Yang C.Q., Shen S., Wu G., Hong W. (2009). Development of self-sensing BFRP bars with distributed optic fiber sensors. Proc. SPIE Int. Soc. Opt. Eng..

[54] Tang Y.S., Wu Z.S., Yang C.Q., Wu G., Shen S. (2010). A new type of smart basalt fiber-reinforced polymer bars as both reinforcements and sensors for civil engineering application. Smart Mater. Struct..

[55] Tang Y.S., Wu Z.S., Yang C.Q., Wu G., Zhao L., Song S. (2010). Application of smart BFRP bars with distributed fiber optic sensors into concrete structures. SPIE Smart Structures & Materials + Nondestructive Evaluation & Health Monitoring.

[56] Luan C.C., Yao X.H., Shen H.Y., Fu J. (2018). Self-Sensing of Position-Related Loads in Continuous Carbon Fibers-Embedded 3D-Printed Polymer Structures Using Electrical Resistance Measurement. Sensors.

[57] Agarwal J., Blockley D., Woodman N. (2003). Vulnerability of structural systems. Struct. Saf..

[58] Cardoso J.B., Almeida J., Dias J.M., Coelho P.G. (2007). Structural reliability analysis using Monte Carlo simulation and neural networks. Adv. Eng. Softw..

[59] Xu H., Rahman S. (2005). Decomposition methods for structural reliability analysis. Probabilistic Eng. Mech..

[60] Elhewy A.H., Mesbahi E., Pu Y. (2006). Reliability Analysis of Structure Using Neural Network method. Probabilistic Eng. Mech..

[61] Hong W., Yang C.Q., Wu Z.S., Zhang Y.F., Wan C., Wu G. (2010). Identification of modal macro-strain vector based on distributed long-gage FBG sensors under ambient vibration. Sensors and Smart Structures Technologies for Civil. Mechanical, and Aerospace Systems.

[62] Zhang J., Xia Q., Cheng Y.Y., Wu Z.S. (2015). Strain flexibility identification of bridges from long-gauge strain measurements. Mech. Syst. Signal Process..

[63] Hong W., Zhang J., Wu G., Wu Z.S. (2015). Comprehensive comparison of macro-strain mode and displacement mode based on different sensing technologies. Mech. Syst. Signal Process..

[64] Ndambi J., Vantomme J., Harri K. (2002). Damage assessment in reinforced concrete beams using eigenfrequencies and mode shape derivatives. Eng. Struct..

[65] Fox C.H. The location of defects in structures: A comparison of the use of natural frequency and mode shape data. Proceedings of the 10th International Modal Analysis Conference.

[66] Osegueda R.A., D’souza P.D., Qiang Y. (1992). Damage evaluation of offshore structures using resonant frequency shifts. Serv. Pet. Process. Power Equip..

[67] Fouad N., Saifeldeen M.A., Huang H., Wu Z.S. (2017). Corrosion monitoring of flexural reinforced concrete members under service loads using distributed long-gauge carbon fiber sensors. Struct. Health Monit..

[68] Zhang J., Cheng Y.Y., Xia Q. (2016). Change localization of a steel-stringer bridge through long-gauge strain measurement. J. Bridge Eng..

[69] Shen S., Wu Z.S., Yang C.Q., Wan C., Tang Y.S., Wu G. (2010). An Improved Conjugated Beam Method for Deformation Monitoring with a Distributed Sensitive Fiber Optic Sensor. Struct. Health Monit..

[70] Zhang J., Tian Y.D., Yang C.Q., Wu B., Wu Z.S., Wu G., Zhang X., Zhou L.M. (2016). Vibration and Deformation Monitoring of a Long-span Rigid-frame Bridge with Distributed Long-gauge Sensors. J. Aerosp. Eng..

[71] Zhang J., Hong W., Tang Y.S., Yang C.Q., Wu G., Wu Z.S. (2014). Structural Health Monitoring of a Steel Stringer Bridge with Area Sensing. Struct. Infrastruct. Eng..

[72] Yang C.Q., Yang D., He Y., Wu Z.S., Xia Y.F., Zhang Y.F. (2015). Moving load identification of small and medium-sized bridges based on distributed optical fiber sensing. Int. J. Struct. Stab. Dyn..

